# Altered expression of the *CSMD1* gene in the peripheral blood of schizophrenia patients

**DOI:** 10.1186/s12888-019-2089-4

**Published:** 2019-04-15

**Authors:** Yansong Liu, Xiaoqian Fu, Zhen Tang, Cui Li, Yong Xu, Fuquan Zhang, Deyi Zhou, Chunming Zhu

**Affiliations:** 1 Department of Clinical Psychology, Suzhou Guangji Hospital, The Affiliated Guangji Hospital of Soochow University, Suzhou, 215137 Jiangsu China; 2 Department of Psychology, Xinghua People’s Hospital, Xinghua, 225700 Jiangsu China; 3 Department of Psychiatry, First Clinical Medical College/First Hospital of Shanxi Medical University, Taiyuan, China; 4 Department of Clinical Psychology, Wuxi Mental Health Center, Nanjing Medical University, 156 Qianrong Road, Wuxi, Jiangsu Province, 214151 China

**Keywords:** Schizophrenia, *CSMD1*, Antipsychotics

## Abstract

**Background:**

Schizophrenia (SCZ) is a heritable, refractory, and devastating psychiatric disorder. Previous studies have shown that the variants of CUB and sushi multiple domains 1 (*CSMD1*) demonstrate significant genome-wide association with SCZ. However, few studies have been conducted on the effect of antipsychotics on the expression levels of *CSMD1*. This study explored whether a change occurs in the expression of the *CSMD1* gene before and after antipsychotic treatment in SCZ patients.

**Methods:**

The study population comprised Han Chinese patients from eastern China, including 32 SCZ patients and 48 healthy controls. The expression of *CSMD1* before and after treatment in the SCZ group and between the two groups was analyzed using real-time quantitative polymerase chain reaction (RT-qPCR).

**Results:**

The expression levels of the *CSMD1* gene in the peripheral blood mononuclear cells (PBMCs) of SCZ patients were lower than those in the healthy controls. The expression levels of the *CSMD1* gene in the PBMCs of the SCZ patients after antipsychotic treatment were higher than those in the baseline SCZ patients (all *P* <  0.05).

**Conclusions:**

Our results showed that the expression levels of *CSMD1* are correlated with the development and treatment of SCZ, providing further evidence for the involvement of *CSMD1* in SCZ.

## Background

Schizophrenia (SCZ) is a hereditary, disabling, and common psychiatric disorder with a worldwide prevalence of approximately 1%. The typical symptoms of SCZ usually appear in youth, with chronic deregulation of thoughts, perceptions, emotions, and behaviors. Genetic factors are the most crucial mechanism in the etiology of SCZ, accounting for approximately 80%; however, these genetic mechanisms remain unclear [1, 2].

The CUB and sushi multiple domains 1 (*CSMD1*) gene is a complement control-related protein located on 8p23.2, the variants of which were reported to have a strong correlation with SCZ risk in a recent study [3]. The single-nucleotide polymorphism (SNP) rs10503253 inside the *CSMD1* gene has been reported to be a major genome-wide risk locus for SCZ [4], and has also been shown to be associated with multiple neurodevelopmental disorders [3, 5, 6]. Studies on SCZ patients of European ancestry have shown that the A risk allele of rs10503253 is related to impaired cognitive function in SCZ [4, 7] and it has been verified as a risk allele of SCZ [8–10]. Moreover, the disruption of *CSMD1* may be related to neuropsychological deficits in csmd1 knockout mice [11]. Furthermore, *CSMD1* has been reported as being involved in regulating the ratio between dopamine and serotonin metabolites in cerebrospinal fluid [12]. Collectively, these findings indicate that the *CSMD1* gene is closely related to the neurogenesis, cognition, immunity, neuropsychology, and monoamine metabolism of SCZ. However, recent studies on the relationship between *CSMD1* and SCZ have been inconsistent; studies have also found no association between rs10503253 and SCZ in patients of Japanese [13] and Han Chinese descent [14].

Although many studies have been conducted on the correlation between *CSMD1* and SCZ, few studies have been conducted on variations in the expression of the *CSMD1* gene. Changes in gene expression may explain the molecular mechanism of SCZ and the efficacy of antipsychotics against it [15]. The alteration of gene expression can be tested in peripheral blood mononuclear cells (PBMCs) because it can be collected from patients more easily than brain tissue. It also helps to identify the characteristics of the clinical subtypes and prognosis of the disease and possible reactions to medication [16]. More importantly, some studies have found that gene expression in the blood and brain overlap considerably [17] and PBMCs have been used in many gene expression analysis studies, as summarized in a recent review [18].

Changes in some gene expressions have been found to be associated with antipsychotic medication for SCZ. Some studies have found that antipsychotic drugs can change the gene expression related to the metabolic pathway in patients with SCZ [19, 20]. Several studies have shown that the expression of some genes is overexpressed, whereas that of others is down-regulated before treatment in SCZ patients, and the use of antipsychotics can partially restore the expression to control levels. Studies have also suggested that these genes are associated with positive symptoms of SCZ based on nearly 95% of patients having a favorable response to drugs [16, 20]. This may indicate that some of the beneficial effects of antipsychotic drugs are mediated by regulation of the expression of these genes.

Although many other gene expression changes have been found to be related to the efficacy of antipsychotic drugs, studies on the effects of antipsychotics on the expression of *CSMD1* in SCZ are largely absent from the worldwide literature. The *CSMD1* gene is closely related to SCZ, and the aforementioned studies indicated that changes in its expression are involved in the molecular mechanism of SCZ and the efficacy of antipsychotics against it. Therefore, whether *CSMD1* gene expression is affected by antipsychotic drug treatment warrants investigation. In this study, we aimed to determine the expression level of the *CSMD1* gene in PBMCs before and after antipsychotic treatment to explore the therapeutic value of *CSMD1* in SCZ.

## Methods

### Patient recruitment

We recruited 32 SCZ patients (17 females and 15 males) and 48 healthy controls (HCs) (31 females and 17 males), all of whom were from a Han Chinese population in Shanxi Province, China. No obvious statistical differences were observed between the SCZ group and the HC group in terms of sex, age, or race (Table 1).

**Table 1:** Demographics of HCs and SCZ patients

Variable	HCs	SCZ	t/χ2	*P* value
Sex (M/F)	17/31	15/17	1.05	0.36
Age (years)	34.06 ± 8.04	37.94 ± 10.30	−1.89	0.16
Ethnicity	Han	Han		

Notes: HCs: healthy controls; SCZ: schizophrenia patients before treatment; M: male; F: female

The case group comprised SCZ patients recruited from the First Hospital of Shanxi Medical University who conformed to the criteria of the Diagnostic and Statistical Manual of Mental Disorders, Fifth Edition (DSM-V), as confirmed by two experienced psychiatrists. Patients with SCZ were not treated with antipsychotic medication before they entered the group. A total of 32 SCZ patients were treated with oral second generation antipsychotics (risperidone (*n* = 5), olanzapine (*n* = 10), quetiapine (*n* = 6), ziprasidone (*n* = 2), aripiprazole (*n* = 6), amisulpride (*n* = 3)) and were followed-up for 12 weeks. Based on the evaluation of a Positive and Negative Syndrome Scale reduction rate of over 25%, the clinical symptoms of the patients in the case group were all improved. The exclusion criteria included severe organic brain injury, mental retardation, epilepsy, alcohol or substance abuse, and other mental disorders.

The HC participants were randomly recruited from local communities in Shanxi Province, and they had no mental or neurological diseases according to a Structured Clinical Interview for DSM-IV and Non-patients. The exclusion criteria included those with a family history of mental illness among first-degree relatives, and those born outside Shanxi Province according to a self-assessment questionnaire. The HC participants and the SCZ patients lived in the same geographic location and were matched for age, sex, and race.

This clinical study was approved by the Ethics Committee of the Wuxi Mental Health Center and the Ethics Committee of the First Hospital of Shanxi Medical University. Informed consent forms were signed by the patients, or by their guardians if they were unable to make their own decisions.

### RNA extraction and analysis of gene expression

A 3-mL sample of peripheral blood was taken from each of the 48 HCs and 32 SCZ patients before and after antipsychotic treatment. Leukocytes were isolated from the fresh blood sample through centrifugation. We extracted total RNA from PBMCs by using TRIzol reagent (Invitrogen, Waltham, MA, USA) with on-column DNase I treatment, as described by the manufacturer. The purity and integrity of total RNA were evaluated using ultraviolet spectrometric measurements and denaturing agarose gel electrophoresis, respectively. A High-Capacity RNA-to-cDNA Kit (Invitrogen) was used to synthesize cDNA, as described by the manufacturer. The expression levels of *CSMD1* in the PBMCs of the 48 HCs and 32 SCZ patients before and after 12-week antipsychotic treatment were measured using real-time quantitative polymerase chain reaction (RT-qPCR) [21], which was performed using a SYBR® Select Master Mix (Invitrogen). PCR was performed using a 7900HT real-time PCR machine (Applied Biosystems, Foster City, CA, USA) for 2 min at 50 °C and 2 min at 95 °C, followed by 40 cycles consisting of 15 s at 95 °C, 60 s at 60 °C. Finally, a standard dissociation protocol was used to ensure that each amplicon was a single product. Each RT-qPCR was performed in triplicate for each of the three independent samples. The expression of the glyceraldehyde-3-phosphate dehydrogenase (GAPDH) gene was used as an internal control and all quantifications were normalized to it. The PCR primers for *CSMD1* were GTCTGGGCTCGTGGATATGT (forward) and CAGGTCTCGGAAGGACAGAG (reverse).

### Statistical analysis

We used the Statistical Package for the Social Sciences 20.0 for statistical analysis. Sex and age were compared between the SCZ and control groups by using the χ^2^ test and an independent-samples *t* test, respectively. The relative expression level of *CSMD1* of each patient after normalization to the glyceraldehyde 3-phosphate dehydrogenase gene was analyzed using the comparative Ct (2^−ΔΔCt^) method. The expression levels of *CSMD1* were compared between the HC and SCZ groups by using the Mann–Whitney U test, and the expression of *CSMD1* was also compared before and after treatment in the SCZ group by using the Mann–Whitney U test. Two-tailed *P* values of less than 0.05 were considered to be statistically significant.

## Results

**Fig. 1:**
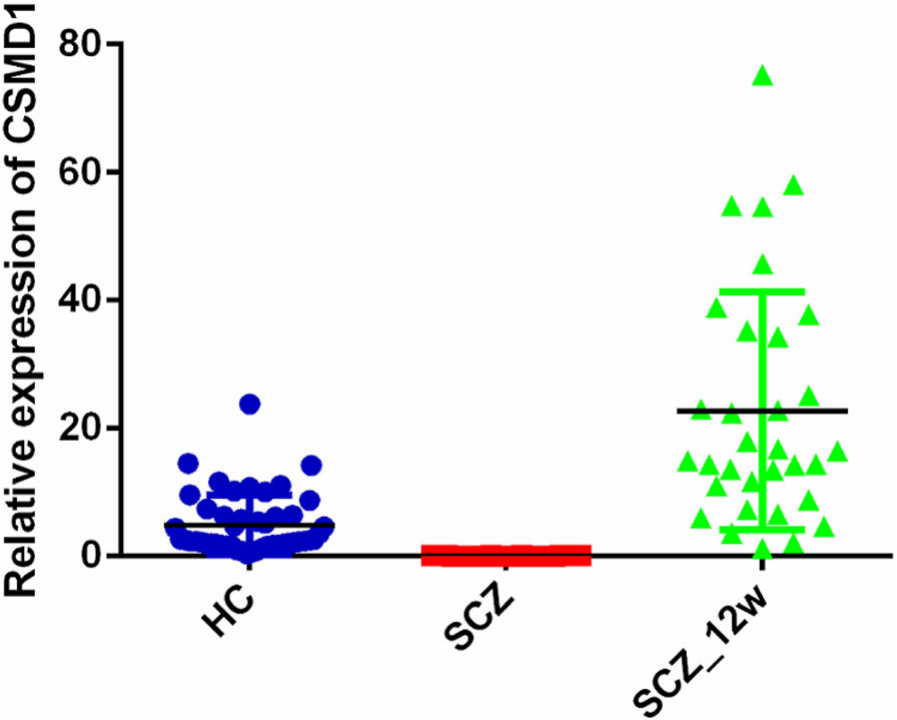
Comparison of *CSMD1* expression levels in the SCZ, SCZ_12w, and HC groups. Notes: HCs: Healthy controls; SCZ: schizophrenia patients before treatment; SCZ_12w: schizophrenia patients after 12 weeks of treatment. ① The expression levels of the *CSMD1* gene in the PBMCs of the SCZ patients were lower than in the HCs. Mann–Whitney U test, *P* < 0.05. ② The expression levels of the *CSMD1* gene in the PBMCs of the SCZ_12w patients were higher than in the baseline SCZ patients. Mann–Whitney U test, *P* < 0.05

The relative expression levels of the *CSMD1* gene in the SCZ patients before treatment, SCZ patients after the 12-week treatment, and the HCs were compared. The results revealed that the expression levels of the *CSMD1* gene in the PBMCs of the SCZ patients were lower than in the HCs (Z = − 7.54, *P* <  0.01). However, after 12 weeks antipsychotic treatment, the expression levels of the *CSMD1* gene in the PBMCs of the SCZ patients were markedly increased (Z = − 6.88, P <  0.01) (Table 2 and Fig. 1).

**Table 2:** Comparison of median *CSMD1* levels in SCZ, SCZ_12w, and HCs

Group	Median (IQR)	Z values	*P* value
HCs (*n* = 48)	2.51 (1.82–6.38)		
SCZ (*n* = 32)	0.05 (0.03–0.08)	−7.54①	**<** 0.01
SCZ_12w (n = 32)	15.75 (9.40–35.01)	−6.88②	**<** 0.01

Notes: HCs: healthy controls; SCZ: schizophrenia patients before treatment; SCZ_12w: schizophrenia patients after 12-weeks of treatment; IQR: interquartile range

① HC vs SCZ; ② SCZ vs SCZ_12w

## Discussion

To the best of our knowledge, this is the first study on changes in the level of *CSMD1* expression in SCZ patients before and after treatment. This study revealed that the expression level of the *CSMD1* gene in PBMCs was up-regulated after antipsychotic treatment (Fig. 1 and Table 2). One mouse study that knocked out the *CSMD1* gene revealed that it may be associated with the internal phenotypes of mental illness, including partial negative symptoms and a dull emotional response in SCZ [11]. Based on the down-regulated expression of *CSMD1* in the SCZ group compared with the HC group and the response of *CSMD1* levels to antipsychotic drugs, we speculate that disruption of the expression of the *CSMD1* gene increases the risk of SCZ. The up-regulation of *CSMD1* was consistent with the improvement of mental symptoms in the SCZ patients after antipsychotic treatment, demonstrating that the change of *CSMD1* expression was attributable to drug therapy. Consequently, our results indicated that the *CSMD1* gene may be a potential biological marker for the treatment of SCZ. Because effects on gene expression are associated with the type of antipsychotic drug [22], changes in *CSMD1* expression may be related to the type of antipsychotic drugs, and their specific relationships warrant further study.

A genome-wide association study [12] on the level of monoamine metabolites in human cerebrospinal fluid revealed that *CSMD1* is related to the regulation of the ratio between dopamine and serotonin metabolites in cerebrospinal fluid. More specifically, the risk allele A of *CSMD1* is associated with a higher rate of dopamine turnover, which is consistent with the high dopamine state of the etiological hypothesis of SCZ [12]. Another study on the molecular mechanism of antipsychotics revealed that many genes can adjust dopamine receptor levels, indicating that changes in gene expression are consistent with known molecular mechanisms of the antipsychotic effect [20]. The dopamine hypothesis plays a critical role in the etiology of SCZ. Most antipsychotics exert an antagonistic effect on dopamine receptors, which may improve psychotic symptoms by blocking the function of dopamine receptors [23, 24]. This study revealed that the *CSMD*1 gene had a favorable response to antipsychotic drug intervention, suggesting that *CSMD1* may be a new dopamine-related gene involved in the molecular mechanism of antipsychotic drugs.

A previous study was conducted to determine the gene expression that can predict the efficacy of antipsychotic drugs, revealing that genes associated with SCZ are generally of higher predictive value [25]. The *CSMD1* gene, the variants of which are strongly correlated with SCZ risk [3], plays a role in preventing complement C3 activation [26, 27]. Studies have indicated that complement components (including complement C3) help the brain develop and function by making precise connections to the synapses and neurons [28, 29]. Abnormality of a complement may affect the risk of neurodegenerative and mental illness by causing abnormal synaptic elimination in the brain [30]. In addition, *CSMD1* has been reported to be the target of MIR137, which has a strong association with SCZ [3] and is involved in the regulation of adult neurogenic and neuronal maturity [31]. In summary, previous studies have shown that *CSMD1* is closely related to SCZ. Our results showed that the *CSMD1* gene has a favorable therapeutic response to antipsychotic drugs, thus proving that *CSMD1* may be a candidate gene for predicting the therapeutic response to antipsychotic drugs.

The *CSMD1* gene is related to immune function through complement regulation, but the precise mechanism remains unclear [26]. The *CSMD1* gene is related to complement regulation and encodes a protein related to complement control through the replication of multiple CUB and sushi domains [11]. *CSMD1* may also play a critical role in regulating complement activation and inflammation in the developing central nervous system [26, 27]. Notably, antipsychotic drugs such as phenothiazine can affect immune-related genes, leading to oxidative stress in the surrounding tissues [22]. For instance, serum C-reactive protein is substantially increased in patients taking typical antipsychotic drugs [22]. However, the exact mechanism of the drug effect on the immunological aspects of *CSMD1* needs further study.

Our research has some limitations. First, the patient population was relatively small, which may have affected the statistical effect in comparing the *CSMD1* gene expression level between the two groups. In the future, larger sample sizes are needed to validate our findings. Second, considering the effects of inflammation or antipsychotic drugs, the same gene may be expressed differently in different cell types. Hence, more accurate cell typing is necessary to study gene expression in the future. Third, a caveat to interpretation of results was that we used only one control gene (GAPDH) for normalization in the RT-qPCR experiments, so the results may be influenced by changes in GAPDH.

## Conclusions

Our results indicated that the expression of *CSMD1* is associated with both the development and antipsychotic treatment of SCZ, suggesting that the *CSMD1* gene may be a promising biological marker for SCZ.

## Abbreviations

*CSMD1*: CUB and sushi multiple domains 1
DSM-IV: Diagnostic and Statistical Manual of Mental Disorders, 4th edition
F: Female
GAPDH: Glyceraldehyde-3-phosphate dehydrogenase
GWAS: Genome-wide association study
HCs: Healthy controls
IQR: Interquartile range
M: Male
RT-qPCR: Real-time quantitative polymerase chain reaction
SCZ: Schizophrenia
SCZ: Schizophrenia patients before medication
SCZ_12w: schizophrenia patients after 12-week medication
SNP: Single-nucleotide polymorphism
